# Does patient’s sex influence treatment in primary care? Experiences and expressed knowledge among physicians – a qualitative study

**DOI:** 10.1186/s12875-015-0351-5

**Published:** 2015-10-13

**Authors:** Desirée Loikas, Linnéa Karlsson, Mia von Euler, Karin Hallgren, Karin Schenck-Gustafsson, Pia Bastholm Rahmner

**Affiliations:** Department of Medicine, Solna Clinical Epidemiology Unit, Karolinska Institutet, Stockholm, Sweden; Department of Healthcare Development, The Health and Medical Care Administration, Stockholm County Council, Stockholm, Sweden; Department of Medicine, Centre for Gender Medicine, Karolinska Institutet and Karolinska University Hospital, Stockholm, Sweden; Department of Clinical Science and Education, Södersjukhuset, Karolinska Institutet Stroke Research Network at Södersjukhuset Karolinska Institutet, Stockholm, Sweden; Department of Clinical Pharmacology, Karolinska Institutet, Karolinska University Hospital, Stockholm, Sweden; Medical Management Centre, Department of Learning, Informatics, Management and Ethics, Karolinska Institutet, Stockholm, Sweden

**Keywords:** Drug utilisation, Female, General practitioners, Male, Sex factors, Qualitative research

## Abstract

**Background:**

Biological and sociocultural differences between men and women may play an important role in medical treatment. Little is known about the awareness of these differences among general practitioners (GPs) and if they consider such differences in their medical practice. The aim of this study was to explore GPs’ perception of sex and gender aspects in medical treatment.

**Methods:**

We conducted five focus group discussions (FGDs) with 29 physicians (mainly GPs) in Sweden. A discussion guide with semi-structured questions was used. All FGDs were audio-recorded and transcribed word-by-word. Data were analysed through inductive thematic analysis with no predetermined categories.

**Results:**

Three main categories emerged from the data. The first category emphasised GPs’ experiences of sex and gender differences in diagnosing and assessment of clinical findings. Medical treatment in men and women was central in the second category. The third category emphasised GPs’ knowledge of sex differences in drug therapy.

**Conclusions:**

The GPs stated they had little knowledge of sex and gender differences in drug treatment, but gave multiple examples of how the patient’s sex affects the choice of treatment. Sex and gender aspects were considered in diagnosing and in the treatment decision. However, once the decision to treat was made the choice of drug followed recommendations by local Drug and Therapeutics Committee, which were perceived to be evidence-based. In the analysis we found a gap between perceived and expressed knowledge of sex and gender differences in drug treatment indicating a need of education about this to be included in the curriculum in medical school and in basic and specialist training for physicians. Education could also be a tool to avoid stereotypical thinking about male and female patients.

## Background

There are biological differences between men and women that may influence medical treatment [1]. Acknowledging this might lead to better health care and treatment outcomes for both men and women. Women are prescribed more medicines than men in most ages, even if hormonal treatments such as contraceptives or hormonal replacement therapy are excluded [2–4]. One reason might be that women have more contact with primary health care [5–7]. There are conflicting results on whether the patient’s sex is associated with delay in diagnosing serious conditions such as malignant and chronic diseases [5, 8, 9]. Health care seeking behaviour differs between men and women due to both sex (biological) and gender (behavioural socioculturally related) differences [7, 10]. As health care consultations often result in a prescription, health care seeking behaviour may in itself influence drug utilisation [11]. Overall, women have been shown to suffer from adverse drug reactions (ADRs) to a higher degree than men [12, 13]. Several drugs have different patterns of adverse effects in men and women [14, 15]. Effective dose may vary as there are pharmacokinetic and pharmacodynamics differences between men and women [16, 17]. Teaching about sex and gender differences in health care seeking patterns, drug utilisation and clinical pharmacology have varied over time and between different medical schools. It is unclear how much general practitioners (GPs) know about these differences and how much attention they pay to them. The aim of this study was to explore GPs’ perception of sex and gender aspects in medical treatment.

## Methods

### Study design

We used a qualitative research approach as this methodology is well suited for studying perceptions and experiences of different phenomenon [18]. Focus group discussions (FGDs) were chosen as data collection method since they are particularly useful when the aim is to gain different views on a specific topic [18–20]. The group process in a FGD drive the informants to concretise ideas and to find mutual experiences, that may not have been expressed in another context [19]. In contrast to a series of individual interviews, participants in a FGD will hear each other’s responses and can thus give additional comments and develop and supplement their answers [19, 20].

### Setting and sample

Since we wanted information-rich cases, the informants were selected by using strategic sample selection [18]. Most health care and medical treatment are carried out by GPs and GPs face patients with a large variety of conditions and diagnoses [21]. The informants were recruited from health centres in different geographic areas in Sweden where the physicians were interested in participating in FGDs about treatment of men and women. These factors were considered to provide a good basis for discussions related to the research questions.

In Sweden, health care is publicly funded and provided by county councils. Swedish GPs work in public or tax-financed private health centres, which are often multidisciplinary with physicians and registered nurses and sometimes also midwives, gynaecologists, physiotherapists and psychologists. In contrast to many other European countries, the GPs do not have a gatekeeper function and the patients are allowed to consult other specialists without a referral. However, the GPs are expected to have the overall responsibility for their patients [22].

### Data collection

The heads of three health centres from different socioeconomic areas in an urban area of Sweden were contacted and informed about the study. All three agreed to participate and were asked to recruit four to eight physicians within their health centre. The FGDs were carried out in the autumn of 2012. The third FGD did not give any new information and thoughts related to the aim of the study. Therefore we performed two additional FGDs to see if there were any other perspectives and ideas among GPs in other, non-urban, areas of Sweden. These two FGDs were carried out in the spring of 2013. However, no additional information was obtained and thus we assumed that saturation was reached. In all, 29 informants, both men and women at different ages participated in the FGDs. By definition, GPs are specialists in family medicine. In our study, most participating physicians were specialised in family medicine but there were also interns and resident physicians among the participants (Table 1). For simplicity reasons, we refer to all physicians who participated in the FGDs as GPs.

**Table 1:** Description of the informants participating in the FGDs

Characteristics	n	%
*Sex*		
Male	12	41
Female	17	59
*Age*		
20–29	1	3
30–39	6	21
40–49	7	24
50–59	8	28
60–69	5	17
70+	1	3
Unknown	1	3
*Position*		
Specialist in family medicine	19	66
Resident	4	14
Intern/Rotation	4	14
Specialist in internal medicine/cardiology	2	7

Two researchers (DL and KH/LK) were present during the FGDs, as moderator and observer. The moderator’s role was to lead the informants in the FGDs, to ensure that they discussed the questions among themselves and not just with the moderator, and that everyone had the opportunity to speak. The observer supported the moderator by asking probing questions [20].

Before the FGD started the informants were informed about the study and that their participation was voluntary and their confidentiality guaranteed. All informants signed a consent form. A semi-structured discussion guide (Table 2) was used throughout the FGDs [18]. The discussion guide was pre-tested to see that no integrity violating questions were used.

**Table 2:** Discussion guide with topics for the FGDs

Introductory question about gender equality and treatment in general
1. How do you reason about gender equality in your work regarding the care of patients?
Questions more specifically about medicines
2. In what way does the patient’s sex affect how you reason about treatment/medication?
3. Do you believe that female and male patients expect different treatment/medication? How/in which way?
4. Can differences in behavior between male and female patients’ result in different treatment/medication?
5. We know that there are certain differences between the sexes where men and women sometimes are treated with different medications in spite of the same diagnosis, and that women receive more medications than men. What are your reflections on this?
6. Do you believe that you as physicians can affect the sex differences in drug utilisation? How/in which way?
Concluding questions
7. How do you perceive your knowledge is regarding this topic?
8. How did you find it to discuss this topic?

### Data analysis

All FGDs were audio-recorded and transcribed word-by-word by two researchers (DL and KH). An inductive thematic analysis with no predetermined categories was performed [18] in a stepwise manner (Table 3).

**Table 3:** Description of the analysis process

1. Tapes, transcripts and field notes from the FGDs were listened to and read repeatedly to get a good grasp of the material.
2. Sections of text in the transcripts, focusing on the research question, were grouped into two main categories; “Sex and gender aspects on patient visit” and “Patient sex in relation to drug treatment”.
3. The sections of text in each main category were summarised and grouped by content into preliminary subcategories.
4. The next step was to find related patterns within each preliminary subcategory. Sections of text were moved between subcategories and new subcategories were formed.
5. The subcategories were grouped into three categories (Table 4).
6. Quotes were selected to illustrate the categories. These quotes were translated to English by the authors. Repetitions and unnecessary words such as humming were removed. To ensure that the sense in the content wasn’t changed in the translation, the quotes were cross translated back to Swedish by a native English speaking person.

### Ethical approval

The study has received ethical approval from the Regional Ethics Committee at Karolinska Institutet, Sweden (ref. no. 2014/2161-31/5).

## Results

Three main categories emerged from the data after analysis. Categories and sub categories are presented below (Table 4).

**Table 4:** Categories and subcategories of Swedish GPs knowledge and views on how patient’s sex influence practice

Category	Subcategory
1. Experiences of sex and gender differences in diagnosing and assessment of clinical findings	a. Sex differences in symptomatology, diseases and morbidity
	b. GPs’ views on different health care seeking behaviour in men and women
	c. Influence of sex and gender on the interaction between GP and patient
2. Medical treatment in men and women	a. Making treatment decisions
	b. GPs’ views on patients’ attitudes to medicines
	c. Adverse drug reactions in men and women
3. Knowledge of sex differences in drug therapy	a. GPs’ expressed knowledge
	b. GPs’ expressed ignorance

### Experience of sex and gender differences in diagnosing and assessment of clinical findings

#### a. Sex differences in symptomatology, diseases and morbidity

The GPs described that patterns of disease differ between men and women. For diagnoses with obvious sex differences such as urinary tract infections, the GPs said they considered the patient’s sex. Some symptom diagnoses such as pain were perceived to be more common in women. Women were described as having more morbidity than men, partly due to reproductive health. The GPs experienced that more women than men needed short- and long-term sick leave.

The GPs stated that presentation of diseases and symptoms may differ between the sexes. Women were perceived to express a more diffuse symptomatology, especially in cardiovascular diseases, and being more difficult to diagnose. In depression, the GPs experienced that women expressed the expected symptoms of being sad while men expressed more aggression and irritability.

The GPs claimed that, as diseases may present differently in men and women and some diagnoses are more common in one sex, the patient’s sex do affect how they examine, decide on a diagnosis and consider differential diagnoses, investigations and treatment.

*That you interpret certain parts of the medical history differently if it concerns a man or a woman. That you deduce the cause differently without really evaluating what they [the patients] say. (Female8 FGD2, in response to the question how lack of awareness of sex and gender could affect diagnosing)*

#### b. GPs’ views on different health care seeking behaviour in men and women

The GPs experienced that women are seeking more health care than men, particularly for urgent medical needs. However, the GPs had the impression that younger men were more inclined to seek health care than older men and that the sex gap in health care seeking behaviour is diminishing. Some GPs reflected on the risk of men receiving less health care than women due to health care seeking behaviour.

*If you consider that men seek [health care] less often, it might be more equal to actively make appointments with men who do not seek health care. But I don’t think about this actively. (Male5 FGD2)*

The GPs believed that women had a lower threshold to seek health care. One suggested explanation was that women are more used to contacts with health care because they take a greater responsibility for the entire family’s health. Another explanation mentioned was that there are more screening programs for women, which will increase the contacts with health care. Men were perceived to have a higher threshold for seeking health care. Some GPs said that men often had been sent there by their mother or partner. A perception of men as hearty, strong and able to look after themselves was expressed.

*Men are big and strong and have a higher threshold to seek health care. I can imagine that there are differences. But it is nothing that we possibly can affect that much. (Female13 FGD3)*

In view of this, the GPs regarded a man seeking for urgent medical needs to be more likely to have a genuine health problem. While some GPs found men to have longer delays in seeking health care, maybe due to ignoring their symptoms in order to not appear weak, others did not have this experience.

*I find myself thinking, “Oh my God, this person has not sought medical attention,” and that is almost always a man who has been having a symptom, of a cancer for example, far too long. I have never experienced an equivalent situation when the patient is female. (Male5 FGD2)*

Men and women were perceived to seek health care due to different causes. GPs perceived that more women made appointments due to problems with depressed mood. There was no consensus on whether depression is more common in women, if men ignore such symptoms or if it is more socially accepted for women to seek medical help for depression. Generally, when a man made an appointment for depressive symptoms he was perceived to have a more severe depression, assumed to have delayed making an appointment, and was thus started on treatment immediately.

*But of course I’m thinking that I should just simply start from how the person feels, not if it is a man or a woman. But I can imagine that I unconsciously would expect a man to be more depressed before he seeks medical care for his depression. And then maybe he would get medication faster compared to a woman. (Male4 FGD2)*

Men were perceived to describe their medical problems briefly and to often seek medical attention for a specified problem while women were perceived to have more diverse problems and to describe them more thoroughly. As men were perceived to delay contact with health care, there was a concern among the GPs that they risked missing morbidity in men.

#### c. Influence of sex on the interaction between GP and patient

According to the GPs, both physician’s and patient’s sex influenced the physician-patient interaction. Some found it easier to handle a patient of their own sex, while others considered personal chemistry and individual factors to be of greater importance. Also, their own prejudices about the patient’s problem were proposed to be a factor in the interaction and that their own sex was perceived to be of importance particularly for patients with sex related disorders. The GPs knew that most nurses, who book the GP appointments, also assumed this and booked accordingly, for example a male patient with a prostate problem was sent to a male GP. Also, patients could request to see a male or female physician. Some GPs believed that the physician’s sex played a less important role for male patients as men are used to being cared for by women.

A belief among the GPs was that there is a difference in how male and female physicians treat patients. They experienced that female GPs attracted patients with more multi-morbidity and social problems as they were expected to be more caring.

Some GPs believed they treated men and women in the same way while others claimed to speak differently and ask different questions to men and women, putting more focus on the family situation with women and on the work situation with men.

*I think we speak differently with men and women, maybe focusing more on family problems and all that with a woman, possibly a bit more than you do with a man, perhaps unconsciously. I think in any case that we speak different “languages” with men and women, perhaps without even thinking about it. (Female13 FGD3)*

### Medical treatment in men and women

#### a. Making treatment decisions

The GPs described that choice of treatment was based both on their own and the patient’s preferences and opinions. They reported that individual factors such as personality had a larger impact than the patient’s sex on the outcome of the visit. The GPs believed that their own sex might influence their view of a patient and their prescribing, but again, that other factors such as experiences and opinions had more influence.

*Yes sex probably affects in some way, the question is how much. I think maybe personality affects more than sex does… (Male8 FGD4)*

On the issue of using pharmacological treatment or not, the GPs believed that there might be differences based on whether the patient is a man or a woman. Less prescribing of analgesics to men as they were considered to be able to endure pain was given as example. Treatment in depressive disorders was given as another example where treatment decision was affected by patient’s sex.

*When thinking about pain you might assume that a man can endure some pain, that you are not as inclined to prescribe painkillers to a man as to a woman. Or maybe you feel a bit more sorry for the woman. (Male8 FGD4)*

When treating more diffuse, unspecific medical conditions in women (*see also1**a. Sex differences in symptomatology, diseases and morbidity**)*, the GPs described that they did not really know what to do. They said they tried to help the patient and offer some treatment or action, often prescribing pills although they were aware of that this was not always the best solution.

*There may be more unexplored diseases affecting women, or, well, more unspecific symptom patterns that we do not really have an understanding of… But it’s very easy when you feel frustrated and want to help that you think, “Well, but there must be some medicine for this”, and there is usually something you can prescribe. (Female17 FGD5)*

The GPs said they followed guidelines and recommendations to a great extent and used drugs from recommendation lists particularly from the Drug and Therapeutics Committees (DTCs). The recommendation list was described as a good guidance and the GPs believed it to consider sex and gender aspects. Some GPs felt limited by the recommendations as they offer a limited choice of drugs.

The GPs said that they don’t take patient’s sex into account when deciding on type of drug therapy. They thought they didn’t make any differences between men and women and that they prescribed the same drug for patients with the same diagnosis. Urinary tract infection was raised as an exception in which the patient’s sex determined the treatment and men and women were treated differently due to biological reasons. Another situation when the patient’s sex became apparent was during pregnancy.

*If I diagnose two patients, a man and a woman, they will get the same treatment regardless of sex. On condition that everything is consistent. But on the road to a diagnosis I might have missed that the woman is little different, or the man is little different, if you know what I mean. When I have come to the conclusion that I should treat someone for a specific disease then the sex is not relevant. (Male1 FGD1)*

The GPs described that there are some physiological differences between men and women they consider when selecting drugs. However, patient’s sex was described as one of several factors to take into account and the GPs held individual factors such as comorbidities and age to be more important.

#### b. GPs’ views on patients’ attitudes to medicines

Male and female patients were perceived to have different expectations of health care and treatment. Women were considered to be more prone to accept suggestions of medicines and more often express a desire to try medicines, including herbals. Some GPs perceived men to have a more negative attitude toward medicines, while some perceived women to be more negative. Others experienced no sex differences in attitude to medicines. According to the GPs, attitudes towards medication use could depend on where in the country you live, particularly if the area is rural or urban. Also ethnicity was raised as a factor that influenced the patient’s view on medicines.

The GPs felt that more women than men have preconceived notions about medications and what they want or don’t want to have. This was thought to be related to women discussing their medications with others more often than men do. Some GPs described that men were more likely to accept the physician’s suggestions while women were more likely to have concerns and want to debate the suggestions.

*There are many women who have said that “my friend had that and it was horrible, I certainly do not want to have it.” I know only one man who has said this. (Male3 FGD2)*

An experience among the GPs was that an increasing number of patients were well informed about diseases and treatments, regardless of the patient’s sex. However, some GPs thought that men used to be better informed than women but that this had changed. Higher level of education was perceived to increase the likelihood of a patient being well informed. To bring clippings from magazines were considered to be typical for women.

*I think it is more associated to profession than to sex. Teachers and engineers, they are the most active in retrieving information. They can carry a whole pile of papers… (Male1 FGD1)*

#### c. Adverse drug reactions in men and women

Some GPs said that they were not aware of sex differences in ADRs and that they did not think it might be different in men and women. The GPs said that they prescribed some drugs to a lesser extent to one sex because of the risk of ADRs. Examples of this were avoidance of beta blockers to men due to risk of impotence or of diuretics to older men not to aggravate prostate problems. The GPs also described that they sometimes preferred a particular drug to a patient of one sex because they wanted to take advantage of some special properties of the drug, for example diuretics to women with hypertension and swollen ankles. Some GPs said that they prescribe drugs according to recommendations but are more observant of potential ADRs in one of the sexes.

*I think that treatment of high blood pressure is what is closest at hand, partly due to the mechanism of action. If you have trouble with water retention in your body, it’s usually a woman, and then thiazides and diuretics may be the drugs of choice. You think of it first because it lowers the blood pressure, reduces the swelling in the body and also has a certain preventive effect on osteoporosis, well doesn’t it sound fine… However, if you are a man about fifty-five to sixty and start taking thiazides, then there is a risk that you may have to start getting up at night because the prostate, that you had no troubles with before, it might start to cause problems. (Female1 FGD1)*

The GPs said they used the same drug doses in men and women. However, some GPs said they prescribed lower doses of certain drugs to women, especially older women, to reduce the risk of ADRs.

*Doses, for example, you might not use as high doses to a woman as to a man, especially if they are elderly. I think women get more adverse effects, statistically speaking, and that is often a matter of dosage. (Female17 FGD5)*

There were different views on sex differences in ADRs. One view was that women have more ADRs than men, while others thought women to report more ADRs. Some GPs felt that women with ADRs talked to their physician while men stopped taking their medication. Regardless of approach, the GPs said that they believed there were sex differences in ADRs for certain substances. Statins and ACE inhibitors were mentioned as examples. The GPs described that there is a risk of misinterpreting symptoms that are more common in one sex as ADRs. Muscle pain was mentioned as an example of a common ADR of statins but also a symptom that is perceived to be more common in women.

Women were perceived to be more sensitive to medicines and often say that they were not able to tolerate certain drugs. The GPs experienced women to want to change treatment because of suspected ADRs more often, requiring several changes before finding a well-tolerated medicine.

*There is a perception that there are more women [than men] who say they do not tolerate certain drugs, and it can be diffuse side effects that we find it difficult to relate to sometimes. (Male9 FGD4)*

### Knowledge of sex differences in drug therapy

#### a. GPs’ expressed knowledge

The GPs discussed the problem of many drugs being poorly studied in women and that evidence on how to treat women is sometimes lacking. The cardiovascular area was considered the most explored. Knowledge of sex differences in newer drugs, such as ACE inhibitors and statins, were considered to be better than for older drugs. The GPs described that they tried to take known sex differences into account when prescribing. Some GPs recalled a lecture in medical school about sex differences in cardiovascular diseases, but otherwise could not recollect receiving any education about sex differences in drug therapy.

*It is exactly at the moment of prescribing you decide on what kind of medicine to choose, regardless if the patient is a man or a woman. I think we [GPs] in general have rather poor knowledge, it’s not just me, I think everyone has. And many drugs are not studied in both men and women. In those cases where I know that there may be sex differences I try to take it into consideration. (Female17 FGD5)*

It was emphasised that there was no evidence that women and men should have identical treatment. However, the GPs said that they assumed that drugs help equally well.

#### b. GPs’ expressed ignorance

The GPs rated their knowledge about sex differences as low. In general, the GPs assumed that men and women should be treated in the same way. They reasoned; if there were differences between men and women in ADRs this would have been noted and treatment recommendations changed. As they treated men and women in the same way there could not be any major differences. If there were large sex differences they would treat differently. A need of more knowledge about pharmacological treatments, where sex differences need to be considered, was expressed.

*So my last question about your knowledge of the topic… (Moderator FGD4) Well as you notice, completely non-existent. (Male9 FGD4) (Laughter) It is low. (Male7 FGD4) You have said that a few times. (Moderator FGD4) Non-existent to low. (Male8 FGD4)*

## Discussion

To our knowledge, this is the first study exploring GPs’ perceptions on sex and gender aspects in medical treatment. Our study do not analyse what physicians actually do, which has been done earlier in studies conducted on how GPs act when treating men and women with standardised symptoms and showing that the sex of the patient influence GPs diagnostic and management activities [23–25]. In our study, the GPs stated they had little knowledge of sex differences in drug treatment. However, they gave several examples of how they considered this in their drug prescription indicating the opposite. The GPs also described sex differences in health care seeking behaviour (i.e. women seeking more health care than men), management (i.e. asking women more about family situation) and morbidity (i.e. different prognosis and presentation of diseases), differences that are in agreement with scientific literature [26]. Most experiences were not perceived by the GPs as proven and thus not perceived as knowledge. In our analysis of the FGDs we find a gap between the GPs perceived knowledge, which was considered low, and the rather extensive examples the GPs gave reflecting actual knowledge and experiences. This gap may indicate that education about sex and gender aspects in medicine has been lacking during their medical training [27] and in their continuing medical education. The lack of education was interpreted by the GPs that there is a lack of evidence-based knowledge or that the issue is of no importance.

The GPs claimed to treat men and women equally, but in other contexts they expressed thoughts that were not based on a gender equality perspective. Stereotypical perceptions of men and women affected the GPs’ thoughts about diseases, diagnosis and differential diagnoses. Physicians making medical decisions based on assumptions and stereotypical thinking has also been shown in other studies [28, 29]. This works as long as patients act as their stereotypes with symptoms and behaviour, but when patients don’t, incorrect decisions about treatment can be made. For example, there may be a risk that the GPs underestimate the emergency level of women’s diseases as well as over-treat men when patients do not seek health care according to their stereotype.

During the FGDs it became obvious that sex and gender considerations were made concerning diagnosis and decision to treat or not. That the sex of the patient influences physicians diagnostic and management activities has been shown in studies from the US and the UK [23, 24]. The GPs in our study stated that once the decision to treat was made, choice of drug followed recommendations from the respective DTC. Since there is a pressure to follow the recommendations from the DTC this is not surprising [30]. However, this is in contrast to the study by Arber where fewer women with coronary heart disease were found to be given appropriate medication [24]. Also in Sweden, women with atrial fibrillation have been treated with anticoagulants to a lower extent than men [31, 32]. The FGDs revealed a high trust in the recommended treatment choices in accordance with a study of attitudes to the recommendations in Stockholm where 81 percent of prescribers found the DTC’s recommendations to be trustworthy. Of interviewed patients 90 percent wanted their doctor to follow the recommendations [30]. The awareness of sex and gender differences for the recommended drugs among the experts making the recommendations has not yet been explored and may vary. While there are sex specific recommendations in some medical areas [33] this is not always the case.

### Strengths and limitations

Given the design of the study, we were only able to get data on what the GPs said they did; not what they actually do in practice. Still, a relationship between what people say and do could be anticipated. To increase the validity between “saying and doing” we frequently used follow-up questions during the FGDs asking for concrete examples of the participants’ way of acting [18]. Furthermore, FGDs are a suitable method to explore unreflected topics, i.e. topics that one do not have thought about or discussed that much [18–20]. Sex and gender issues in drug treatment turned out to be an unreflected topic. The method with FGDs where the informants interact and help each other to explore and clarify their views, ways that would be less easily accessible in individual interviews, was thus preferable. Another strength of this study was the participation of GPs with varying degrees of professional experience, working in different geographic and socioeconomic areas of Sweden. Furthermore, the GPs did not seem to have any hesitations about expressing conflicting opinions. Otherwise, it can be a risk in FGDs that the participants modify their answers either to please the researcher or to avoid conflicts with other participants [20]. Nevertheless, these five FGDs gave a rich and nuanced picture of GPs’ views on sex differences related to drug treatment.

## Conclusions

In this study of physicians (mainly GPs) working in primary care settings in different parts of Sweden, the physicians stated they had little knowledge of sex and gender differences in drug treatment. Nevertheless, they gave multiple examples of how the patient’s sex affects the choice of treatment. The FGDs showed that GPs considered sex and gender in diagnosing and deciding to treat or not. However, once the decision to treat was made, choice of drug followed recommendations from the respective Drug and Therapeutics Committee as they were believed to be evidence based and sex neutral. To bridge the gap between perceived and expressed awareness, education on sex and gender differences in drug treatment needs to be included in the curriculum in medical school and in basic and specialist training for physicians. Education could also be a tool to avoid stereotypical thinking about male and female patients.

## Abbreviations

GP: General practitioner
FGD: Focus group discussion
ADR: Adverse drug reaction
DTC: Drug and Therapeutics Committee
